# Supplementary material for: A fault-intrusion-tolerant system and deadline-aware algorithm for scheduling scientific workflow in the cloud
Source: PeerJ Comput Sci. 2021 Nov 2;7:e747. doi: 10.7717/peerj-cs.747 (PMC8576554; doi:10.7717/peerj-cs.747)
Supplement: Supplemental Information 6 [file peerj-cs-07-747-s006.zip › Myproject1.1/docs/index-files/index-7.html]

G-Index


JavaScript is disabled on your browser.


- Overview
- Package
- Class
- Use
- Tree
- Deprecated
- Index
- Help

- Prev Letter
- Next Letter

- Frames
- No Frames

- All Classes

A B C D E F G H I J L M N O P Q R S T U V W Z 


## G

GammaDistr - Class in org.cloudbus.cloudsim.distributions
:   The Class GammaDistr.

GammaDistr(Random, int, double) - Constructor for class org.cloudbus.cloudsim.distributions.GammaDistr
:   Instantiates a new gamma distr.

GammaDistr(int, double) - Constructor for class org.cloudbus.cloudsim.distributions.GammaDistr
:   Instantiates a new gamma distr.

generate(Job) - Static method in class org.workflowsim.failure.FailureGenerator
:   Generates a failure or not

generateWorkload() - Method in class org.cloudbus.cloudsim.util.WorkloadFileReader
:   Reads job information from a given file.

generateWorkload() - Method in interface org.cloudbus.cloudsim.util.WorkloadModel
:   Returns a list with the jobs generated by the workload.

getActualCPUTime(int) - Method in class org.cloudbus.cloudsim.Cloudlet
:   Gets the total execution time of this Cloudlet in a given CloudResource ID.

getActualCPUTime() - Method in class org.cloudbus.cloudsim.Cloudlet
:   Returns the time the Cloudlet actually run.

getAdvReservList() - Method in class org.cloudbus.cloudsim.core.CloudInformationService
:   Gets the list of CloudResource IDs that **only** support Advanced Reservation.

getAllocatedBwForVm(Vm) - Method in class org.cloudbus.cloudsim.provisioners.BwProvisioner
:   Gets the allocated BW for VM.

getAllocatedBwForVm(Vm) - Method in class org.cloudbus.cloudsim.provisioners.BwProvisionerSimple


getAllocatedMips() - Method in class org.cloudbus.cloudsim.HostStateHistoryEntry
:   Gets the allocated mips.

getAllocatedMips() - Method in class org.cloudbus.cloudsim.VmStateHistoryEntry
:   Gets the allocated mips.

getAllocatedMipsForVm(Vm) - Method in class org.cloudbus.cloudsim.Host
:   Returns the MIPS share of each Pe that is allocated to a given VM.

getAllocatedMipsForVm(Vm) - Method in class org.cloudbus.cloudsim.provisioners.PeProvisioner
:   Gets allocated MIPS for a given VM.

getAllocatedMipsForVm(Vm) - Method in class org.cloudbus.cloudsim.provisioners.PeProvisionerSimple


getAllocatedMipsForVm(Vm) - Method in class org.cloudbus.cloudsim.VmScheduler
:   Returns the MIPS share of each Pe that is allocated to a given VM.

getAllocatedMipsForVmByVirtualPeId(Vm, int) - Method in class org.cloudbus.cloudsim.provisioners.PeProvisioner
:   Gets allocated MIPS for a given VM for a given virtual Pe.

getAllocatedMipsForVmByVirtualPeId(Vm, int) - Method in class org.cloudbus.cloudsim.provisioners.PeProvisionerSimple


getAllocatedRamForVm(Vm) - Method in class org.cloudbus.cloudsim.provisioners.RamProvisioner
:   Gets the allocated RAM for VM.

getAllocatedRamForVm(Vm) - Method in class org.cloudbus.cloudsim.provisioners.RamProvisionerSimple


getAllocationPolicy() - Method in class org.cloudbus.cloudsim.DatacenterCharacteristics
:   Gets the allocation policy.

getAllResourceId() - Method in class org.cloudbus.cloudsim.Cloudlet
:   Gets all the CloudResource IDs that executed this Cloudlet.

getAllResourceName() - Method in class org.cloudbus.cloudsim.Cloudlet
:   Gets all the CloudResource names that executed this Cloudlet.

getAllVmList() - Method in class org.workflowsim.WorkflowEngine


getAlpha() - Static method in class org.workflowsim.utils.Parameters
:   Gets the task failure rate

getAppCloudletList() - Method in class org.cloudbus.cloudsim.network.datacenter.NetDatacenterBroker


getArchitecture() - Method in class org.cloudbus.cloudsim.DatacenterCharacteristics
:   Gets the architecture.

getArrayList() - Method in class org.workflowsim.clustering.AbstractArrayList
:   Gets the task list

getAttributeSize() - Method in class org.cloudbus.cloudsim.File
:   Gets the size of this object (in byte).  
    NOTE: This object size is NOT the actual file size.

getAttributeSize() - Method in class org.cloudbus.cloudsim.FileAttribute
:   Gets the size of this object (in byte).  
    NOTE: This object size is NOT the actual file size.

getAvailableBw() - Method in class org.cloudbus.cloudsim.provisioners.BwProvisioner
:   Gets the available BW in the host.

getAvailableMips() - Method in class org.cloudbus.cloudsim.Host
:   Gets the free mips.

getAvailableMips() - Method in class org.cloudbus.cloudsim.provisioners.PeProvisioner
:   Gets the available MIPS in the PE.

getAvailableMips() - Method in class org.cloudbus.cloudsim.VmScheduler
:   Gets the free mips.

getAvailableRam() - Method in class org.cloudbus.cloudsim.provisioners.RamProvisioner
:   Gets the available RAM in the host.

getAvailableSpace() - Method in class org.cloudbus.cloudsim.HarddriveStorage
:   Gets the available space on this storage in MB.

getAvailableSpace() - Method in interface org.cloudbus.cloudsim.Storage
:   Gets the available space on this storage in MByte.

getAvgSeekTime() - Method in class org.cloudbus.cloudsim.HarddriveStorage
:   Gets the average seek time of the harddrive in seconds.

getBandwidth() - Method in class org.workflowsim.utils.OverheadParameters
:   Gets the bandwidth

getBaudRate() - Method in class org.cloudbus.cloudsim.InfoPacket
:   Returns the bottleneck bandwidth between the source and the destination.

getBeta() - Static method in class org.workflowsim.utils.Parameters
:   Gets the job failure rate (not supported yet)

getBroker() - Method in class org.cloudbus.cloudsim.examples.CloudSimExample8.GlobalBroker


getBw() - Method in class org.cloudbus.cloudsim.Host
:   Gets the machine bw.

getBw() - Method in class org.cloudbus.cloudsim.provisioners.BwProvisioner
:   Gets the bw.

getBw() - Method in class org.cloudbus.cloudsim.Vm
:   Gets the amount of bandwidth.

getBwProvisioner() - Method in class org.cloudbus.cloudsim.Host
:   Gets the bw provisioner.

getBwTable() - Method in class org.cloudbus.cloudsim.provisioners.BwProvisionerSimple
:   Gets the bw table.

getById(List<T>, int) - Static method in class org.cloudbus.cloudsim.lists.CloudletList
:   Gets the by id.

getById(List<T>, int) - Static method in class org.cloudbus.cloudsim.lists.HostList
:   Gets the Machine object for a particular ID.

getById(List<T>, int) - Static method in class org.cloudbus.cloudsim.lists.PeList
:   Gets MIPS Rating for a specified Pe ID.

getById(List<T>, int) - Static method in class org.cloudbus.cloudsim.lists.VmList
:   Return a reference to a Vm object from its ID.

getByIdAndUserId(List<T>, int, int) - Static method in class org.cloudbus.cloudsim.lists.ResCloudletList
:   Returns a given Cloudlet.

getByIdAndUserId(List<T>, int, int) - Static method in class org.cloudbus.cloudsim.lists.VmList
:   Return a reference to a Vm object from its ID and user ID.

getCacheCurrentRequestedMips() - Method in class org.cloudbus.cloudsim.CloudletSchedulerDynamicWorkload
:   Gets the cache current requested mips.

getCachePreviousTime() - Method in class org.cloudbus.cloudsim.CloudletSchedulerDynamicWorkload
:   Gets the cache previous time.

getCandidateTastSet(ArrayList<TaskSet>, TaskSet, int) - Method in class org.workflowsim.clustering.balancing.methods.HorizontalDistanceBalancing
:   Gets the potential candidate taskSets to merge

getCandidateTastSet(ArrayList<TaskSet>, TaskSet, int) - Method in class org.workflowsim.clustering.balancing.methods.HorizontalImpactBalancing
:   Gets the potential candidate taskSets to merge

getCapacity(List<Double>) - Method in class org.cloudbus.cloudsim.CloudletSchedulerTimeShared
:   Gets the capacity.

getCapacity() - Method in class org.cloudbus.cloudsim.HarddriveStorage
:   Gets the total capacity of the storage in MB.

getCapacity() - Method in interface org.cloudbus.cloudsim.Storage
:   Gets the total capacity of the storage in MByte.

getCharacteristics() - Method in class org.cloudbus.cloudsim.Datacenter
:   Gets the characteristics.

getChecksum() - Method in class org.cloudbus.cloudsim.File
:   Gets the file checksum

getChecksum() - Method in class org.cloudbus.cloudsim.FileAttribute
:   Gets the file checksum

getChildList() - Method in class org.workflowsim.clustering.TaskSet
:   Gets the child list

getChildList() - Method in class org.workflowsim.Task
:   Gets the child list of the task

getClassType() - Method in class org.cloudbus.cloudsim.Cloudlet
:   Gets the classtype or priority of this Cloudlet for scheduling on a resource.

getClouddletFinishTime() - Method in class org.cloudbus.cloudsim.ResCloudlet
:   Gets the Cloudlet's finish time.

getCloudInfoServiceEntityId() - Static method in class org.cloudbus.cloudsim.core.CloudSim
:   Gets the entity ID of CloudInformationService.

getCloudlet() - Method in class org.cloudbus.cloudsim.ResCloudlet
:   Gets this Cloudlet object.

getCloudletArrivalTime() - Method in class org.cloudbus.cloudsim.ResCloudlet
:   Gets arrival time of a cloudlet.

getCloudletClassType() - Method in class org.cloudbus.cloudsim.ResCloudlet
:   Gets the Cloudlet's class type.

getCloudletExecList() - Method in class org.cloudbus.cloudsim.CloudletSchedulerSpaceShared
:   Gets the cloudlet exec list.

getCloudletExecList() - Method in class org.cloudbus.cloudsim.CloudletSchedulerTimeShared
:   Gets the cloudlet exec list.

getCloudletExecList() - Method in class org.cloudbus.cloudsim.network.datacenter.NetworkCloudletSpaceSharedScheduler
:   Gets the cloudlet exec list.

getCloudletFileSize() - Method in class org.cloudbus.cloudsim.Cloudlet
:   Gets the input file size of this Cloudlet BEFORE submitting to a CloudResource.

getCloudletFinishedList() - Method in class org.cloudbus.cloudsim.CloudletSchedulerSpaceShared
:   Gets the cloudlet finished list.

getCloudletFinishedList() - Method in class org.cloudbus.cloudsim.CloudletSchedulerTimeShared
:   Gets the cloudlet finished list.

getCloudletFinishedList() - Method in class org.cloudbus.cloudsim.network.datacenter.NetworkCloudletSpaceSharedScheduler
:   Gets the cloudlet finished list.

getCloudletFinishedSoFar() - Method in class org.cloudbus.cloudsim.Cloudlet
:   Gets the length of this Cloudlet that has been executed so far from the latest CloudResource.

getCloudletFinishedSoFar(int) - Method in class org.cloudbus.cloudsim.Cloudlet
:   Gets the length of this Cloudlet that has been executed so far in a given CloudResource ID.

getCloudletHistory() - Method in class org.cloudbus.cloudsim.Cloudlet
:   Gets the history of this Cloudlet.

getCloudletId() - Method in class org.cloudbus.cloudsim.Cloudlet
:   Gets the ID of this Cloudlet.

getCloudletId() - Method in class org.cloudbus.cloudsim.ResCloudlet
:   Gets this Cloudlet entity Id.

getCloudletLength() - Method in class org.cloudbus.cloudsim.Cloudlet
:   Gets the length of this Cloudlet.

getCloudletLength() - Method in class org.cloudbus.cloudsim.ResCloudlet
:   Gets the Cloudlet's length.

getCloudletList() - Method in class org.cloudbus.cloudsim.DatacenterBroker
:   Gets the cloudlet list.

getCloudletList() - Method in class org.cloudbus.cloudsim.examples.CloudSimExample8.GlobalBroker


getCloudletList() - Method in class org.cloudbus.cloudsim.network.datacenter.NetDatacenterBroker
:   Gets the cloudlet list.

getCloudletList() - Method in class org.workflowsim.scheduler.BaseScheduler
:   Gets the job list.

getCloudletList() - Method in interface org.workflowsim.scheduler.SchedulerInterface
:   Gets the job list.

getCloudletOutputSize() - Method in class org.cloudbus.cloudsim.Cloudlet
:   Gets the output size of this Cloudlet AFTER submitting and executing to a
    CloudResource.

getCloudletPausedList() - Method in class org.cloudbus.cloudsim.CloudletSchedulerSpaceShared
:   Gets the cloudlet paused list.

getCloudletPausedList() - Method in class org.cloudbus.cloudsim.CloudletSchedulerTimeShared
:   Gets the cloudlet paused list.

getCloudletPausedList() - Method in class org.cloudbus.cloudsim.network.datacenter.NetworkCloudletSpaceSharedScheduler
:   Gets the cloudlet paused list.

getCloudletReceivedList() - Method in class org.cloudbus.cloudsim.DatacenterBroker
:   Gets the cloudlet received list.

getCloudletReceivedList() - Method in class org.cloudbus.cloudsim.network.datacenter.NetDatacenterBroker
:   Gets the cloudlet received list.

getCloudletScheduler() - Method in class org.cloudbus.cloudsim.Vm
:   Gets the vm scheduler.

getCloudletStatus() - Method in class org.cloudbus.cloudsim.Cloudlet
:   Gets the status code of this Cloudlet.

getCloudletStatus(int) - Method in class org.cloudbus.cloudsim.CloudletScheduler
:   Gets the status of a cloudlet.

getCloudletStatus(int) - Method in class org.cloudbus.cloudsim.CloudletSchedulerSpaceShared
:   Gets the status of a cloudlet.

getCloudletStatus(int) - Method in class org.cloudbus.cloudsim.CloudletSchedulerTimeShared
:   Gets the status of a cloudlet.

getCloudletStatus(int) - Method in class org.cloudbus.cloudsim.network.datacenter.NetworkCloudletSpaceSharedScheduler
:   Gets the status of a cloudlet.

getCloudletStatus() - Method in class org.cloudbus.cloudsim.ResCloudlet
:   Gets the Cloudlet status.

getCloudletStatusString() - Method in class org.cloudbus.cloudsim.Cloudlet
:   Gets the string representation of the current Cloudlet status code.

getCloudletSubmitted() - Method in class org.cloudbus.cloudsim.power.PowerDatacenter
:   Checks if is cloudlet submited.

getCloudletSubmittedList() - Method in class org.cloudbus.cloudsim.DatacenterBroker
:   Gets the cloudlet submitted list.

getCloudletSubmittedList() - Method in class org.cloudbus.cloudsim.network.datacenter.NetDatacenterBroker
:   Gets the cloudlet submitted list.

getCloudletTotalLength() - Method in class org.cloudbus.cloudsim.Cloudlet
:   Gets the total length (across all PEs) of this Cloudlet.

getCloudletTotalLength() - Method in class org.cloudbus.cloudsim.ResCloudlet
:   Gets the total Cloudlet's length (across all PEs).

getCloudletWaitingList() - Method in class org.cloudbus.cloudsim.CloudletSchedulerSpaceShared
:   Gets the cloudlet waiting list.

getCloudletWaitingList() - Method in class org.cloudbus.cloudsim.network.datacenter.NetworkCloudletSpaceSharedScheduler
:   Gets the cloudlet waiting list.

getCloudResourceList() - Static method in class org.cloudbus.cloudsim.core.CloudSim
:   Sends a request to Cloud Information Service (GIS) entity to get the list of all Cloud
    hostList.

getClustDelay() - Method in class org.workflowsim.utils.OverheadParameters
:   Gets the clustering delay

getClustDelay(Cloudlet) - Method in class org.workflowsim.utils.OverheadParameters
:   Gets the clustering delay for a particular job based on the depth(level)

getClusteringEngine() - Method in class org.workflowsim.WorkflowPlanner
:   Gets the clustering engine

getClusteringEngineId() - Method in class org.workflowsim.WorkflowPlanner
:   Gets the clustering engine id

getClusteringFactor(FailureRecord) - Static method in class org.workflowsim.failure.FailureMonitor
:   Gets the clustering factor

getClusteringMethod() - Method in class org.workflowsim.utils.ClusteringParameters
:   Gets the clustering method

getClusteringParameters() - Static method in class org.workflowsim.utils.Parameters
:   Gets the clustering parameters

getClusterNum() - Method in class org.workflowsim.clustering.balancing.methods.BalancingMethod
:   Gets clusters.num

getClustersNum() - Method in class org.workflowsim.utils.ClusteringParameters
:   Gets the number of clustered jobs per level

getClustersSize() - Method in class org.workflowsim.utils.ClusteringParameters
:   Gets the size of clustered jobs, which is equal to the number of tasks in
    a job

getCode() - Method in class org.workflowsim.utils.ClusteringParameters
:   Gets the code for balanced clustering Please refer to our balanced
    clustering paper for details

getCompletedVms() - Method in class org.cloudbus.cloudsim.HostDynamicWorkload
:   Gets the completed vms.

getConstant() - Method in class org.cloudbus.cloudsim.power.models.PowerModelCubic
:   Gets the constant.

getConstant() - Method in class org.cloudbus.cloudsim.power.models.PowerModelLinear
:   Gets the constant.

getConstant() - Method in class org.cloudbus.cloudsim.power.models.PowerModelSqrt
:   Gets the constant.

getConstant() - Method in class org.cloudbus.cloudsim.power.models.PowerModelSquare
:   Gets the constant.

getCoordinateX() - Method in class org.cloudbus.cloudsim.network.TopologicalNode
:   returns the x coordinate of this network-node

getCoordinateY() - Method in class org.cloudbus.cloudsim.network.TopologicalNode
:   returns the y coordinate of this network-node

getCorrelationCoefficients(double[][]) - Method in class org.cloudbus.cloudsim.power.PowerVmSelectionPolicyMaximumCorrelation
:   Gets the correlation coefficients.

getCost() - Method in class org.cloudbus.cloudsim.File
:   Gets the cost associated with this file

getCost() - Method in class org.cloudbus.cloudsim.FileAttribute
:   Gets the cost associated with this file

getCostPerBw() - Method in class org.cloudbus.cloudsim.DatacenterCharacteristics
:   Get the cost to use bandwidth in this resource.

getCostPerMem() - Method in class org.cloudbus.cloudsim.DatacenterCharacteristics
:   Get the cost to use memory in this resource.

getCostPerMi() - Method in class org.cloudbus.cloudsim.DatacenterCharacteristics
:   Gets the cost per Millions Instruction (MI) associated with a resource.

getCostPerSec() - Method in class org.cloudbus.cloudsim.Cloudlet
:   Gets the cost running this Cloudlet in the latest CloudResource.

getCostPerSec(int) - Method in class org.cloudbus.cloudsim.Cloudlet
:   Gets the cost running this Cloudlet in a given CloudResource ID.

getCostPerSecond() - Method in class org.cloudbus.cloudsim.DatacenterCharacteristics
:   Gets the cost per second.

getCostPerStorage() - Method in class org.cloudbus.cloudsim.DatacenterCharacteristics
:   Get the cost to use storage in this resource.

getCpuTime(double, double) - Method in class org.cloudbus.cloudsim.DatacenterCharacteristics
:   Gets the CPU time given the specified parameters (only for TIME\_SHARED).

getCreationTime() - Method in class org.cloudbus.cloudsim.File
:   Gets the file creation time (in millisecond)

getCreationTime() - Method in class org.cloudbus.cloudsim.FileAttribute
:   Gets the file creation time (in millisecond)

getCurrentAllocatedBw() - Method in class org.cloudbus.cloudsim.Vm
:   Gets the current allocated bw.

getCurrentAllocatedMips() - Method in class org.cloudbus.cloudsim.Vm
:   Gets the current allocated mips.

getCurrentAllocatedRam() - Method in class org.cloudbus.cloudsim.Vm
:   Gets the current allocated ram.

getCurrentAllocatedSize() - Method in class org.cloudbus.cloudsim.Vm
:   Gets the current allocated size.

getCurrentMipsShare() - Method in class org.cloudbus.cloudsim.CloudletScheduler
:   Gets the current mips share.

getCurrentRequestedBw() - Method in class org.cloudbus.cloudsim.Vm
:   Gets the current requested bw.

getCurrentRequestedMaxMips() - Method in class org.cloudbus.cloudsim.Vm
:   Gets the current requested max mips among all virtual PEs.

getCurrentRequestedMips() - Method in class org.cloudbus.cloudsim.CloudletScheduler
:   Gets the current requested mips.

getCurrentRequestedMips() - Method in class org.cloudbus.cloudsim.CloudletSchedulerDynamicWorkload
:   Gets the current mips.

getCurrentRequestedMips() - Method in class org.cloudbus.cloudsim.CloudletSchedulerSpaceShared


getCurrentRequestedMips() - Method in class org.cloudbus.cloudsim.CloudletSchedulerTimeShared


getCurrentRequestedMips() - Method in class org.cloudbus.cloudsim.network.datacenter.NetworkCloudletSpaceSharedScheduler


getCurrentRequestedMips() - Method in class org.cloudbus.cloudsim.Vm
:   Gets the current requested mips.

getCurrentRequestedRam() - Method in class org.cloudbus.cloudsim.Vm
:   Gets the current requested ram.

getCurrentRequestedTotalMips() - Method in class org.cloudbus.cloudsim.Vm
:   Gets the current requested total mips.

getCurrentRequestedUtilizationOfBw() - Method in class org.cloudbus.cloudsim.CloudletScheduler
:   Gets the current requested bw.

getCurrentRequestedUtilizationOfBw() - Method in class org.cloudbus.cloudsim.CloudletSchedulerSpaceShared


getCurrentRequestedUtilizationOfBw() - Method in class org.cloudbus.cloudsim.CloudletSchedulerTimeShared


getCurrentRequestedUtilizationOfBw() - Method in class org.cloudbus.cloudsim.network.datacenter.NetworkCloudletSpaceSharedScheduler


getCurrentRequestedUtilizationOfRam() - Method in class org.cloudbus.cloudsim.CloudletScheduler
:   Gets the current requested ram.

getCurrentRequestedUtilizationOfRam() - Method in class org.cloudbus.cloudsim.CloudletSchedulerSpaceShared


getCurrentRequestedUtilizationOfRam() - Method in class org.cloudbus.cloudsim.CloudletSchedulerTimeShared


getCurrentRequestedUtilizationOfRam() - Method in class org.cloudbus.cloudsim.network.datacenter.NetworkCloudletSpaceSharedScheduler


getCurrentSize() - Method in class org.cloudbus.cloudsim.HarddriveStorage
:   Gets the current size of the stored files in MB.

getCurrentSize() - Method in interface org.cloudbus.cloudsim.Storage
:   Gets the current size of the storage in MByte.

getCurrentTaskSetAtLevels() - Method in class org.workflowsim.clustering.balancing.BalancedClustering
:   Gets the current tasks per level

getData() - Method in class org.cloudbus.cloudsim.core.SimEvent
:   Get the data passed in this event.

getDatacenter() - Method in class org.cloudbus.cloudsim.Host
:   Gets the data center.

getDatacenterCharacteristicsList() - Method in class org.cloudbus.cloudsim.DatacenterBroker
:   Gets the datacenter characteristics list.

getDatacenterCharacteristicsList() - Method in class org.cloudbus.cloudsim.network.datacenter.NetDatacenterBroker
:   Gets the datacenter characteristics list.

getDatacenterIdsList() - Method in class org.cloudbus.cloudsim.DatacenterBroker
:   Gets the datacenter ids list.

getDatacenterIdsList() - Method in class org.cloudbus.cloudsim.network.datacenter.NetDatacenterBroker
:   Gets the datacenter ids list.

getDatacenterRequestedIdsList() - Method in class org.cloudbus.cloudsim.DatacenterBroker
:   Gets the datacenter requested ids list.

getDatacenterRequestedIdsList() - Method in class org.cloudbus.cloudsim.network.datacenter.NetDatacenterBroker
:   Gets the datacenter requested ids list.

getDatasizePath() - Static method in class org.workflowsim.utils.Parameters
:   Gets the data size path

getDaxPath() - Static method in class org.workflowsim.utils.Parameters
:   Gets the DAX path

getDeadline() - Static method in class org.workflowsim.utils.Parameters


getDelay(int, int) - Method in class org.cloudbus.cloudsim.network.DelayMatrix\_Float


getDelay(int, int) - Static method in class org.cloudbus.cloudsim.NetworkTopology
:   Calculates the delay between two nodes

getDepth() - Method in class org.workflowsim.clustering.AbstractArrayList
:   Gets the depth of these tasks

getDepth() - Method in class org.workflowsim.Task
:   Gets the depth of the task

getDestId() - Method in class org.cloudbus.cloudsim.InfoPacket
:   Gets the id of the entity to which the packet is destined.

getDestId() - Method in interface org.cloudbus.cloudsim.Packet
:   Returns the destination id of this packet.

getDestination() - Method in class org.cloudbus.cloudsim.core.SimEvent
:   Get the unique id number of the entity which received this event.

getDestNodeID() - Method in class org.cloudbus.cloudsim.network.TopologicalLink
:   return the node-ID from the DestNode

getDetailBaudRate() - Method in class org.cloudbus.cloudsim.InfoPacket
:   Returns the list of all the bandwidths that this packet has traversed.

getDetailEntryTimes() - Method in class org.cloudbus.cloudsim.InfoPacket
:   Returns the list of all entry time that this packet has traversed.

getDetailExitTimes() - Method in class org.cloudbus.cloudsim.InfoPacket
:   Returns the list of all exit time that this packet has traversed.

getDetailHops() - Method in class org.cloudbus.cloudsim.InfoPacket
:   Returns the list of all the hops that this packet has traversed.

getDurationTime() - Method in class org.cloudbus.cloudsim.ResCloudlet
:   Gets the reservation duration time.

getEdgeSwitch() - Method in class org.cloudbus.cloudsim.network.datacenter.NetworkDatacenter
:   Get list of all EdgeSwitches in the Datacenter network One can design similar functions for
    other type of switches.

getEnergyLinearInterpolation(double, double, double) - Method in class org.cloudbus.cloudsim.power.PowerHost
:   Gets the energy consumption using linear interpolation of the utilization change.

getEntity(int) - Static method in class org.cloudbus.cloudsim.core.CloudSim
:   Get the entity with a given id.

getEntity(String) - Static method in class org.cloudbus.cloudsim.core.CloudSim
:   Get the entity with a given name.

getEntityId(String) - Static method in class org.cloudbus.cloudsim.core.CloudSim
:   Get the id of an entity with a given name.

getEntityList() - Static method in class org.cloudbus.cloudsim.core.CloudSim
:   Returns a list of entities created for the simulation.

getEntityName(int) - Static method in class org.cloudbus.cloudsim.core.CloudSim
:   Gets name of the entity given its entity ID.

getEntityName(Integer) - Static method in class org.cloudbus.cloudsim.core.CloudSim
:   Gets name of the entity given its entity ID.

getEstimatedFinishTime(ResCloudlet, double) - Method in class org.cloudbus.cloudsim.CloudletSchedulerDynamicWorkload
:   Get estimated cloudlet completion time.

getEventBuffer() - Method in class org.cloudbus.cloudsim.core.SimEntity
:   Gets the event buffer.

getExecStartTime() - Method in class org.cloudbus.cloudsim.Cloudlet
:   Gets the latest execution start time.

getExecStartTime() - Method in class org.cloudbus.cloudsim.ResCloudlet
:   Gets the Cloudlet's execution start time.

getExecutionTimeHistoryHostSelection() - Method in class org.cloudbus.cloudsim.power.PowerVmAllocationPolicyMigrationAbstract
:   Gets the execution time history host selection.

getExecutionTimeHistoryTotal() - Method in class org.cloudbus.cloudsim.power.PowerVmAllocationPolicyMigrationAbstract
:   Gets the execution time history total.

getExecutionTimeHistoryVmReallocation() - Method in class org.cloudbus.cloudsim.power.PowerVmAllocationPolicyMigrationAbstract
:   Gets the execution time history vm reallocation.

getExecutionTimeHistoryVmSelection() - Method in class org.cloudbus.cloudsim.power.PowerVmAllocationPolicyMigrationAbstract
:   Gets the execution time history vm selection.

getExecutionTimes() - Static method in class org.cloudbus.cloudsim.util.ExecutionTimeMeasurer
:   Gets the execution times.

getExperimentName(String...) - Method in class org.cloudbus.cloudsim.examples.power.RunnerAbstract
:   Gets the experiment name.

getFailureGeneratorMode() - Static method in class org.workflowsim.utils.Parameters
:   Gets the failure generation mode

getFallbackPolicy() - Method in class org.cloudbus.cloudsim.power.PowerVmSelectionPolicyMaximumCorrelation
:   Gets the fallback policy.

getFallbackVmAllocationPolicy() - Method in class org.cloudbus.cloudsim.power.PowerVmAllocationPolicyMigrationInterQuartileRange
:   Gets the fallback vm allocation policy.

getFallbackVmAllocationPolicy() - Method in class org.cloudbus.cloudsim.power.PowerVmAllocationPolicyMigrationLocalRegression
:   Gets the fallback vm allocation policy.

getFallbackVmAllocationPolicy() - Method in class org.cloudbus.cloudsim.power.PowerVmAllocationPolicyMigrationMedianAbsoluteDeviation
:   Gets the fallback vm allocation policy.

getFile(String) - Method in class org.cloudbus.cloudsim.HarddriveStorage
:   Gets the file with the specified name.

getFile(String) - Method in interface org.cloudbus.cloudsim.Storage
:   Gets the file with the specified name.

getFile(String) - Static method in class org.workflowsim.utils.ReplicaCatalog
:   Gets the file object based its file name

getFileAttribute() - Method in class org.cloudbus.cloudsim.File
:   Gets an attribute of this file

getFileList() - Method in class org.workflowsim.Task
:   Gets the list of the files

getFileNameList() - Method in class org.cloudbus.cloudsim.HarddriveStorage
:   Gets the list of file names located on this storage.

getFileNameList() - Method in interface org.cloudbus.cloudsim.Storage
:   Gets the list of file names located on this storage.

getFileSize() - Method in class org.cloudbus.cloudsim.FileAttribute
:   Gets the file size (in MBytes)

getFileSizeInByte() - Method in class org.cloudbus.cloudsim.FileAttribute
:   Gets the file size (in bytes)

getFileSystem() - Static method in class org.workflowsim.utils.ReplicaCatalog
:   Gets the file system

getFinishTime() - Method in class org.cloudbus.cloudsim.Cloudlet
:   Gets the finish time of this Cloudlet in a CloudResource.

getfreehostlist(int) - Method in class org.cloudbus.cloudsim.network.datacenter.Switch


getFreePe(List<T>) - Static method in class org.cloudbus.cloudsim.lists.PeList
:   Gets a Pe ID which is FREE.

getFreePes() - Method in class org.cloudbus.cloudsim.network.datacenter.NetworkVmAllocationPolicy
:   Gets the free pes.

getFreePes() - Method in class org.cloudbus.cloudsim.VmAllocationPolicySimple
:   Gets the free pes.

getFreePes() - Method in class org.cloudbus.cloudsim.VmSchedulerSpaceShared
:   Gets the free pes vector.

getfreeVmlist(int) - Method in class org.cloudbus.cloudsim.network.datacenter.Switch


getFTCMethod() - Static method in class org.workflowsim.utils.Parameters
:   Gets the fault tolerant clustering method

getHistory() - Method in class org.cloudbus.cloudsim.UtilizationModelStochastic
:   Gets the history.

getHost(Vm) - Method in class org.cloudbus.cloudsim.network.datacenter.NetworkVmAllocationPolicy
:   Gets the host that is executing the given VM belonging to the given user.

getHost(int, int) - Method in class org.cloudbus.cloudsim.network.datacenter.NetworkVmAllocationPolicy
:   Gets the host that is executing the given VM belonging to the given user.

getHost(Vm) - Method in class org.cloudbus.cloudsim.power.PowerVmAllocationPolicyAbstract


getHost(int, int) - Method in class org.cloudbus.cloudsim.power.PowerVmAllocationPolicyAbstract


getHost() - Method in class org.cloudbus.cloudsim.Vm
:   Gets the host.

getHost(Vm) - Method in class org.cloudbus.cloudsim.VmAllocationPolicy
:   Get the host that is executing the given VM belonging to the given user.

getHost(int, int) - Method in class org.cloudbus.cloudsim.VmAllocationPolicy
:   Get the host that is executing the given VM belonging to the given user.

getHost(Vm) - Method in class org.cloudbus.cloudsim.VmAllocationPolicySimple
:   Gets the host that is executing the given VM belonging to the given user.

getHost(int, int) - Method in class org.cloudbus.cloudsim.VmAllocationPolicySimple
:   Gets the host that is executing the given VM belonging to the given user.

getHostList() - Method in class org.cloudbus.cloudsim.Datacenter
:   Gets the host list.

getHostList() - Method in class org.cloudbus.cloudsim.DatacenterCharacteristics
:   Gets the host list.

getHostList() - Method in class org.cloudbus.cloudsim.VmAllocationPolicy
:   Gets the host list.

getHostUtilizationIqr(PowerHostUtilizationHistory) - Method in class org.cloudbus.cloudsim.power.PowerVmAllocationPolicyMigrationInterQuartileRange
:   Gets the host utilization iqr.

getHostUtilizationMad(PowerHostUtilizationHistory) - Method in class org.cloudbus.cloudsim.power.PowerVmAllocationPolicyMigrationMedianAbsoluteDeviation
:   Gets the host utilization mad.

getHostWithFreePe() - Method in class org.cloudbus.cloudsim.DatacenterCharacteristics
:   Gets a Machine with at least one empty Pe.

getHostWithFreePe(int) - Method in class org.cloudbus.cloudsim.DatacenterCharacteristics
:   Gets a Machine with at least a given number of free Pe.

getHostWithFreePe(List<T>) - Static method in class org.cloudbus.cloudsim.lists.HostList
:   Gets a Machine with free Pe.

getHostWithFreePe(List<T>, int) - Static method in class org.cloudbus.cloudsim.lists.HostList
:   Gets a Machine with a specified number of free Pe.

getHostwithVM(int) - Method in class org.cloudbus.cloudsim.network.datacenter.Switch


getId() - Method in class org.cloudbus.cloudsim.core.SimEntity
:   Get the unique id number assigned to this entity.

getId() - Method in class org.cloudbus.cloudsim.DatacenterCharacteristics
:   Gets the id.

getId() - Method in class org.cloudbus.cloudsim.Host
:   Gets the id.

getId() - Method in class org.cloudbus.cloudsim.InfoPacket
:   Returns the ID of this packet.

getId() - Method in interface org.cloudbus.cloudsim.Packet
:   Returns the ID of this packet

getId() - Method in class org.cloudbus.cloudsim.Pe
:   Gets the id.

getId() - Method in class org.cloudbus.cloudsim.Vm
:   Gets the id.

getImpact() - Method in class org.workflowsim.Task
:   Gets the impact of the task

getImpactFactor() - Method in class org.workflowsim.clustering.TaskSet
:   Gets the impact factor

getJobList() - Method in class org.workflowsim.clustering.BasicClustering
:   Gets the job list

getJobList() - Method in interface org.workflowsim.clustering.ClusteringInterface
:   get job list.

getJobList() - Method in class org.workflowsim.ClusteringEngine
:   Gets the job list.

getJobRuntime() - Method in class org.workflowsim.clustering.TaskSet
:   Gets the job runtime of this taskSet (sum of task runtime)

getJobsList() - Method in class org.workflowsim.WorkflowEngine
:   Gets the job list.

getJobsReceivedList() - Method in class org.workflowsim.WorkflowEngine
:   Gets the job received list.

getJobsSubmittedList() - Method in class org.workflowsim.WorkflowEngine
:   Gets the job submitted list.

getK(double, double, double) - Static method in class org.workflowsim.failure.FailureMonitor
:   Gets the optimal clustering factor based on analysis

getLast() - Method in class org.cloudbus.cloudsim.InfoPacket
:   Gets an entity ID from the last hop that this packet has traversed.

getLast() - Method in interface org.cloudbus.cloudsim.Packet
:   Gets an entity ID from the last hop that this packet has traversed.

getLastProcessTime() - Method in class org.cloudbus.cloudsim.Datacenter
:   Gets the last process time.

getLastUpdateTime() - Method in class org.cloudbus.cloudsim.File
:   Gets the last update time (in seconds)

getLastUpdateTime() - Method in class org.cloudbus.cloudsim.FileAttribute
:   Gets the last update time (in seconds)

getLatency() - Method in class org.cloudbus.cloudsim.HarddriveStorage
:   Gets the latency of this harddrive in seconds.

getLevelMap() - Method in class org.workflowsim.clustering.balancing.methods.BalancingMethod
:   Gets the level map

getLinkBw() - Method in class org.cloudbus.cloudsim.network.TopologicalLink
:   return the link-bw of the defined linke

getLinkDelay() - Method in class org.cloudbus.cloudsim.network.TopologicalLink
:   return the link-delay of the defined linke

getLinkIterator() - Method in class org.cloudbus.cloudsim.network.TopologicalGraph
:   return an iterator through all network-graph links

getList() - Method in class org.cloudbus.cloudsim.core.CloudInformationService
:   Gets the list of all CloudResource IDs, including hostList that support Advance Reservation.

getLoessParameterEstimates(double[]) - Static method in class org.cloudbus.cloudsim.util.MathUtil
:   Gets the loess parameter estimates.

getMachineId() - Method in class org.cloudbus.cloudsim.ResCloudlet
:   Gets machine ID.

getMachineIdList() - Method in class org.cloudbus.cloudsim.ResCloudlet
:   Gets a list of Machine IDs.

getMaxAvailableMips() - Method in class org.cloudbus.cloudsim.Host
:   Returns maximum available MIPS among all the PEs.

getMaxAvailableMips() - Method in class org.cloudbus.cloudsim.VmScheduler
:   Returns maximum available MIPS among all the PEs.

getMaxAvailableMips() - Method in class org.cloudbus.cloudsim.VmSchedulerTimeShared
:   Returns maximum available MIPS among all the PEs.

getMaximumVmMigrationTime(PowerHost) - Method in class org.cloudbus.cloudsim.power.PowerVmAllocationPolicyMigrationLocalRegression
:   Gets the maximum vm migration time.

getMaxPower() - Method in class org.cloudbus.cloudsim.power.models.PowerModelCubic
:   Gets the max power.

getMaxPower() - Method in class org.cloudbus.cloudsim.power.models.PowerModelLinear
:   Gets the max power.

getMaxPower() - Method in class org.cloudbus.cloudsim.power.models.PowerModelSqrt
:   Gets the max power.

getMaxPower() - Method in class org.cloudbus.cloudsim.power.models.PowerModelSquare
:   Gets the max power.

getMaxPower() - Method in class org.cloudbus.cloudsim.power.PowerHost
:   Gets the max power that can be consumed by the host.

getMaxTransferRate() - Method in class org.cloudbus.cloudsim.HarddriveStorage
:   Gets the maximum transfer rate of the storage in MB/sec.

getMaxTransferRate() - Method in class org.cloudbus.cloudsim.SanStorage
:   Gets the maximum transfer rate of the storage in MB/sec.

getMaxTransferRate() - Method in interface org.cloudbus.cloudsim.Storage
:   Gets the maximum transfer rate of the storage in MByte/sec.

getMaxTransferRate(String) - Method in class org.workflowsim.ClusterStorage
:   Gets the bandwidth from this storage to the destination storage

getMaxUtilization() - Method in class org.cloudbus.cloudsim.HostDynamicWorkload
:   Gets the max utilization among by all PEs.

getMaxUtilization(List<T>) - Static method in class org.cloudbus.cloudsim.lists.PeList
:   Gets the max utilization among by all PEs.

getMaxUtilizationAfterAllocation(NetworkHost, Vm) - Method in class org.cloudbus.cloudsim.network.datacenter.NetworkVmAllocationPolicy


getMaxUtilizationAfterAllocation(PowerHost, Vm) - Method in class org.cloudbus.cloudsim.power.PowerVmAllocationPolicyMigrationAbstract
:   Gets the power after allocation.

getMaxUtilizationAmongVmsPes(Vm) - Method in class org.cloudbus.cloudsim.HostDynamicWorkload
:   Gets the max utilization among by all PEs allocated to the VM.

getMaxUtilizationAmongVmsPes(List<T>, Vm) - Static method in class org.cloudbus.cloudsim.lists.PeList
:   Gets the max utilization among by all PEs allocated to the VM.

getMaxUtilizationAmongVmsPes(Vm) - Method in class org.cloudbus.cloudsim.network.datacenter.NetworkHost


getMetric(ArrayList<TaskSet>) - Method in interface org.workflowsim.clustering.balancing.metrics.BalancingMetric
:   Gets the metric value.

getMetric(ArrayList<TaskSet>) - Method in class org.workflowsim.clustering.balancing.metrics.DistanceVariance
:   Returns the distance variance of a list of taskSets

getMetric(ArrayList<TaskSet>) - Method in class org.workflowsim.clustering.balancing.metrics.HorizontalRuntimeVariance
:   Returns the standard deviation of runtime

getMetric(ArrayList<TaskSet>) - Method in class org.workflowsim.clustering.balancing.metrics.ImpactFactorVariance
:   Returns the standard deviation of their impact factors

getMetric(ArrayList<TaskSet>) - Method in class org.workflowsim.clustering.balancing.metrics.PipelineRuntimeVariance
:   Returns the standard deviation of the pipeline runtime

getMetricHistory() - Method in class org.cloudbus.cloudsim.power.PowerVmAllocationPolicyMigrationAbstract
:   Gets the metric history.

getMigratableVms(PowerHost) - Method in class org.cloudbus.cloudsim.power.PowerVmSelectionPolicy
:   Gets the migratable vms.

getMigrationCount() - Method in class org.cloudbus.cloudsim.power.PowerDatacenter
:   Gets the migration count.

getMigrationMapFromUnderUtilizedHosts(List<PowerHostUtilizationHistory>) - Method in class org.cloudbus.cloudsim.power.PowerVmAllocationPolicyMigrationAbstract
:   Gets the migration map from under utilized hosts.

getMinTimeBetweenEvents() - Static method in class org.cloudbus.cloudsim.core.CloudSim
:   Returns the minimum time between events.

getMinUtilizationHistorySize(List<PowerVm>) - Method in class org.cloudbus.cloudsim.power.PowerVmSelectionPolicyMaximumCorrelation
:   Gets the min utilization history size.

getMips() - Method in class org.cloudbus.cloudsim.CloudletSchedulerDynamicWorkload
:   Gets the mips.

getMips() - Method in class org.cloudbus.cloudsim.DatacenterCharacteristics
:   Gets the total MIPS rating, which is the sum of MIPS rating of all machines in a resource.

getMips(List<T>, int) - Static method in class org.cloudbus.cloudsim.lists.PeList
:   Gets MIPS Rating for a specified Pe ID.

getMips() - Method in class org.cloudbus.cloudsim.Pe
:   Gets the MIPS Rating of this Pe.

getMips() - Method in class org.cloudbus.cloudsim.provisioners.PeProvisioner
:   Gets the MIPS.

getMips() - Method in class org.cloudbus.cloudsim.Vm
:   Gets the mips.

getMipsMap() - Method in class org.cloudbus.cloudsim.VmScheduler
:   Gets the mips map.

getMipsMapRequested() - Method in class org.cloudbus.cloudsim.VmSchedulerTimeShared
:   Gets the mips map requested.

getMipsOfOnePe() - Method in class org.cloudbus.cloudsim.DatacenterCharacteristics
:   Gets Millions Instructions Per Second (MIPS) Rating of a Processing Element (Pe).

getMipsOfOnePe(int, int) - Method in class org.cloudbus.cloudsim.DatacenterCharacteristics
:   Gets Millions Instructions Per Second (MIPS) Rating of a Processing Element (Pe).

getMonitorMode() - Static method in class org.workflowsim.utils.Parameters
:   Gets the fault tolerant clustering monitor mode

getName() - Method in class org.cloudbus.cloudsim.core.SimEntity
:   Get the name of this entity.

getName() - Method in class org.cloudbus.cloudsim.File
:   Returns the file name

getName() - Method in class org.cloudbus.cloudsim.FileAttribute
:   Returns the file name

getName() - Method in class org.cloudbus.cloudsim.HarddriveStorage
:   Gets the name of the storage.

getName() - Method in interface org.cloudbus.cloudsim.Storage
:   Gets the name of the storage.

getNetServiceLevel() - Method in class org.cloudbus.cloudsim.Cloudlet
:   Gets the network service level for sending this cloudlet over a network.

getNetServiceType() - Method in class org.cloudbus.cloudsim.InfoPacket
:   Gets the network service type of this packet.

getNetServiceType() - Method in interface org.cloudbus.cloudsim.Packet
:   Gets the network service type of this packet

getNewVmPlacement(List<? extends Vm>, Set<? extends Host>) - Method in class org.cloudbus.cloudsim.power.PowerVmAllocationPolicyMigrationAbstract
:   Gets the new vm placement.

getNewVmPlacementFromUnderUtilizedHost(List<? extends Vm>, Set<? extends Host>) - Method in class org.cloudbus.cloudsim.power.PowerVmAllocationPolicyMigrationAbstract
:   Gets the new vm placement from under utilized host.

getNextEvent(Predicate) - Method in class org.cloudbus.cloudsim.core.SimEntity
:   Get the first event matching a predicate from the deferred queue, or if none match, wait for
    a matching event to arrive.

getNextEvent() - Method in class org.cloudbus.cloudsim.core.SimEntity
:   Get the first event waiting in the entity's deferred queue, or if there are none, wait for an
    event to arrive.

getNextFinishedCloudlet() - Method in class org.cloudbus.cloudsim.CloudletScheduler
:   Returns the next cloudlet in the finished list, $null if this list is empty.

getNextFinishedCloudlet() - Method in class org.cloudbus.cloudsim.CloudletSchedulerSpaceShared
:   Returns the next cloudlet in the finished list, $null if this list is empty.

getNextFinishedCloudlet() - Method in class org.cloudbus.cloudsim.CloudletSchedulerTimeShared
:   Returns the next cloudlet in the finished list, $null if this list is empty.

getNextFinishedCloudlet() - Method in class org.cloudbus.cloudsim.network.datacenter.NetworkCloudletSpaceSharedScheduler
:   Returns the next cloudlet in the finished list, $null if this list is empty.

getNodeID() - Method in class org.cloudbus.cloudsim.network.TopologicalNode
:   delivers the nodes id

getNodeIterator() - Method in class org.cloudbus.cloudsim.network.TopologicalGraph
:   returns an iterator through all network-graph nodes

getNodeLabel() - Method in class org.cloudbus.cloudsim.network.TopologicalNode
:   delivers the name of the node

getNumberOfBusyPes() - Method in class org.cloudbus.cloudsim.DatacenterCharacteristics
:   Gets the total number of BUSY PEs for all Machines.

getNumberOfBusyPes(List<T>) - Static method in class org.cloudbus.cloudsim.lists.HostList
:   Gets the total number of BUSY PEs for all Machines.

getNumberOfBusyPes(List<T>) - Static method in class org.cloudbus.cloudsim.lists.PeList
:   Gets the number of BUSY Pe.

getNumberOfFailedHosts() - Method in class org.cloudbus.cloudsim.DatacenterCharacteristics
:   Gets the current number of failed machines.

getNumberOfFreePes() - Method in class org.cloudbus.cloudsim.DatacenterCharacteristics
:   Gets the total number of FREE or non-busy PEs for all Machines.

getNumberOfFreePes() - Method in class org.cloudbus.cloudsim.Host
:   Gets the free pes number.

getNumberOfFreePes(List<T>) - Static method in class org.cloudbus.cloudsim.lists.HostList
:   Gets the total number of FREE or non-busy PEs for all Machines.

getNumberOfFreePes(List<T>) - Static method in class org.cloudbus.cloudsim.lists.PeList
:   Gets the number of FREE or non-busy Pe.

getNumberOfHosts() - Method in class org.cloudbus.cloudsim.DatacenterCharacteristics
:   Gets the total number of machines.

getNumberOfLinks() - Method in class org.cloudbus.cloudsim.network.TopologicalGraph
:   returns the number of links contained inside the topological-graph

getNumberOfNodes() - Method in class org.cloudbus.cloudsim.network.TopologicalGraph
:   returns the number of nodes contained inside the topological-graph

getNumberOfPes() - Method in class org.cloudbus.cloudsim.Cloudlet
:   Gets the number of PEs required to run this Cloudlet.

getNumberOfPes() - Method in class org.cloudbus.cloudsim.CloudletSchedulerDynamicWorkload
:   Gets the pes number.

getNumberOfPes() - Method in class org.cloudbus.cloudsim.DatacenterCharacteristics
:   Gets the total number of PEs for all Machines.

getNumberOfPes() - Method in class org.cloudbus.cloudsim.Host
:   Gets the pes number.

getNumberOfPes(List<T>) - Static method in class org.cloudbus.cloudsim.lists.HostList
:   Gets the total number of PEs for all Machines.

getNumberOfPes() - Method in class org.cloudbus.cloudsim.ResCloudlet
:   Gets the number of PEs required to execute this Cloudlet.

getNumberOfPes() - Method in class org.cloudbus.cloudsim.Vm
:   Gets the number of pes.

getNumEntities() - Static method in class org.cloudbus.cloudsim.core.CloudSim
:   Get the current number of entities in the simulation.

getNumHop() - Method in class org.cloudbus.cloudsim.InfoPacket
:   Returns the number of hops that this packet has traversed.

getNumStoredFile() - Method in class org.cloudbus.cloudsim.HarddriveStorage
:   Gets the number of files stored on this storage.

getNumStoredFile() - Method in interface org.cloudbus.cloudsim.Storage
:   Gets the number of files stored on this storage.

getnumvm() - Method in class org.cloudbus.cloudsim.examples.network.datacenter.TestBagofTaskApp
:   One can generate number of VMs for each application based on deadline

getOriginalPingSize() - Method in class org.cloudbus.cloudsim.InfoPacket
:   Gets original size of ping request.

getOs() - Method in class org.cloudbus.cloudsim.DatacenterCharacteristics
:   Gets the os.

getOutput() - Static method in class org.cloudbus.cloudsim.Log
:   Gets the output.

getOverheadParams() - Static method in class org.workflowsim.utils.Parameters
:   Gets the overhead parameters

getOverUtilizedHosts() - Method in class org.cloudbus.cloudsim.power.PowerVmAllocationPolicyMigrationAbstract
:   Gets the over utilized hosts.

getOwnerName() - Method in class org.cloudbus.cloudsim.File
:   Gets the owner name of this file

getOwnerName() - Method in class org.cloudbus.cloudsim.FileAttribute
:   Gets the owner name of this file

getParameterEstimates(double[]) - Method in class org.cloudbus.cloudsim.power.PowerVmAllocationPolicyMigrationLocalRegression
:   Gets the parameter estimates.

getParameterEstimates(double[]) - Method in class org.cloudbus.cloudsim.power.PowerVmAllocationPolicyMigrationLocalRegressionRobust
:   Gets the parameter estimates.

getParentList() - Method in class org.workflowsim.clustering.TaskSet
:   Gets the parent list

getParentList() - Method in class org.workflowsim.Job
:   Gets the list of the parent tasks and override its super function

getParentList() - Method in class org.workflowsim.Task
:   Gets the list of the parent tasks

getPeAllocationMap() - Method in class org.cloudbus.cloudsim.VmSchedulerSpaceShared
:   Gets the pe allocation map.

getPeCapacity() - Method in class org.cloudbus.cloudsim.VmScheduler
:   Returns PE capacity in MIPS.

getPeId() - Method in class org.cloudbus.cloudsim.ResCloudlet
:   Gets Pe ID.

getPeIdList() - Method in class org.cloudbus.cloudsim.ResCloudlet
:   Gets a list of Pe IDs.

getPeList() - Method in class org.cloudbus.cloudsim.Host
:   Gets the pe list.

getPeList() - Method in class org.cloudbus.cloudsim.VmScheduler
:   Gets the vm list.

getPeMap() - Method in class org.cloudbus.cloudsim.VmScheduler
:   Gets the pe map.

getPeProvisioner() - Method in class org.cloudbus.cloudsim.Pe
:   Gets the Pe provisioner.

getPesAllocatedForVM(Vm) - Method in class org.cloudbus.cloudsim.VmScheduler
:   Gets the pes allocated for vm.

getPesInUse() - Method in class org.cloudbus.cloudsim.VmSchedulerTimeShared
:   Gets the pes in use.

getPeTable() - Method in class org.cloudbus.cloudsim.provisioners.PeProvisionerSimple
:   Gets the pe table.

getPK() - Method in class org.cloudbus.cloudsim.network.FloydWarshall\_Float
:   gets predecessor matrix

getPlannerMode() - Static method in class org.workflowsim.utils.Parameters
:   Gets the planning method

getPositionById(List<T>, int) - Static method in class org.cloudbus.cloudsim.lists.CloudletList
:   Returns the position of the cloudlet with that id, if it exists.

getPositionById(List<T>, int) - Static method in class org.cloudbus.cloudsim.lists.ResCloudletList
:   Returns the position of the cloudlet with that id, if it exists.

getPostDelay() - Method in class org.workflowsim.utils.OverheadParameters
:   Gets the postscript delay

getPostDelay(Job) - Method in class org.workflowsim.utils.OverheadParameters
:   Gets the postscript delay for a particular job based on the depth(level)

getPower(double) - Method in interface org.cloudbus.cloudsim.power.models.PowerModel
:   Get power consumption by the utilization percentage according to the power model.

getPower(double) - Method in class org.cloudbus.cloudsim.power.models.PowerModelCubic


getPower(double) - Method in class org.cloudbus.cloudsim.power.models.PowerModelLinear


getPower(double) - Method in class org.cloudbus.cloudsim.power.models.PowerModelSpecPower


getPower(double) - Method in class org.cloudbus.cloudsim.power.models.PowerModelSqrt


getPower(double) - Method in class org.cloudbus.cloudsim.power.models.PowerModelSquare


getPower() - Method in class org.cloudbus.cloudsim.power.PowerDatacenter
:   Gets the power.

getPower() - Method in class org.cloudbus.cloudsim.power.PowerHost
:   Gets the power.

getPower(double) - Method in class org.cloudbus.cloudsim.power.PowerHost
:   Gets the power.

getPowerAfterAllocation(PowerHost, Vm) - Method in class org.cloudbus.cloudsim.power.PowerVmAllocationPolicyMigrationAbstract
:   Gets the power after allocation.

getPowerData(int) - Method in class org.cloudbus.cloudsim.power.models.PowerModelSpecPower
:   Gets the power data.

getPowerData(int) - Method in class org.cloudbus.cloudsim.power.models.PowerModelSpecPowerHpProLiantMl110G3PentiumD930


getPowerData(int) - Method in class org.cloudbus.cloudsim.power.models.PowerModelSpecPowerHpProLiantMl110G4Xeon3040


getPowerData(int) - Method in class org.cloudbus.cloudsim.power.models.PowerModelSpecPowerHpProLiantMl110G5Xeon3075


getPowerData(int) - Method in class org.cloudbus.cloudsim.power.models.PowerModelSpecPowerIbmX3250XeonX3470


getPowerData(int) - Method in class org.cloudbus.cloudsim.power.models.PowerModelSpecPowerIbmX3250XeonX3480


getPowerData(int) - Method in class org.cloudbus.cloudsim.power.models.PowerModelSpecPowerIbmX3550XeonX5670


getPowerData(int) - Method in class org.cloudbus.cloudsim.power.models.PowerModelSpecPowerIbmX3550XeonX5675


getPowerModel() - Method in class org.cloudbus.cloudsim.power.PowerHost
:   Gets the power model.

getPreviousTime() - Method in class org.cloudbus.cloudsim.CloudletScheduler
:   Gets the previous time.

getPreviousTime() - Method in class org.cloudbus.cloudsim.power.PowerVm
:   Gets the previous time.

getPreviousUtilizationMips() - Method in class org.cloudbus.cloudsim.HostDynamicWorkload
:   Gets the previous utilization mips.

getPreviousUtilizationOfCpu() - Method in class org.cloudbus.cloudsim.HostDynamicWorkload
:   Gets the previous utilization of CPU in percentage.

getPriority() - Method in class org.workflowsim.Task
:   Gets the priority of the task

getProcessingCost() - Method in class org.cloudbus.cloudsim.Cloudlet
:   Gets the total cost of processing or executing this Cloudlet
    Processing Cost = input data transfer + processing cost + output transfer cost .

getQueueDelay() - Method in class org.workflowsim.utils.OverheadParameters
:   Gets the queue delay

getQueueDelay(Cloudlet) - Method in class org.workflowsim.utils.OverheadParameters
:   Gets the queue delay for a particular job based on the depth(level)

getRam() - Method in class org.cloudbus.cloudsim.Host
:   Gets the machine memory.

getRam() - Method in class org.cloudbus.cloudsim.provisioners.RamProvisioner
:   Gets the ram.

getRam() - Method in class org.cloudbus.cloudsim.Vm
:   Gets the amount of ram.

getRamProvisioner() - Method in class org.cloudbus.cloudsim.Host
:   Gets the ram provisioner.

getRamTable() - Method in class org.cloudbus.cloudsim.provisioners.RamProvisionerSimple
:   Gets the ram table.

getRandomGenerator() - Method in class org.cloudbus.cloudsim.UtilizationModelStochastic
:   Gets the random generator.

getReduceMethod() - Static method in class org.workflowsim.utils.Parameters
:   Gets the reducer mode

getRegionalCisName() - Method in class org.cloudbus.cloudsim.Datacenter
:   Gets the regional cis name.

getRegistrationID() - Method in class org.cloudbus.cloudsim.File
:   Gets the file registration ID

getRegistrationID() - Method in class org.cloudbus.cloudsim.FileAttribute
:   Gets the file registration ID

getRemainingCloudletLength() - Method in class org.cloudbus.cloudsim.ResCloudlet
:   Gets the remaining cloudlet length.

getRequestedMips() - Method in class org.cloudbus.cloudsim.HostStateHistoryEntry
:   Gets the requested mips.

getRequestedMips() - Method in class org.cloudbus.cloudsim.VmStateHistoryEntry
:   Gets the requested mips.

getRequiredFiles() - Method in class org.cloudbus.cloudsim.Cloudlet
:   Gets the required files.

getReservationId() - Method in class org.cloudbus.cloudsim.Cloudlet
:   Gets the reservation ID that owns this Cloudlet.

getReservationID() - Method in class org.cloudbus.cloudsim.ResCloudlet
:   Gets the reservation ID that owns this Cloudlet.

getResourceById(int) - Method in class org.cloudbus.cloudsim.Cloudlet
:   Gets the resource by id.

getResourceId() - Method in class org.cloudbus.cloudsim.Cloudlet
:   Gets the latest resource ID that processes this Cloudlet.

getResourceID() - Method in class org.cloudbus.cloudsim.File
:   Gets the resource ID that stores this file

getResourceID() - Method in class org.cloudbus.cloudsim.FileAttribute
:   Gets the resource ID that stores this file

getResourceName(int) - Method in class org.cloudbus.cloudsim.Cloudlet
:   Gets the CloudResource name based on its ID.

getResourceName() - Method in class org.cloudbus.cloudsim.DatacenterCharacteristics
:   Gets the name of a resource.

getRobustLoessParameterEstimates(double[]) - Static method in class org.cloudbus.cloudsim.util.MathUtil
:   Gets the robust loess parameter estimates.

getRuntimePath() - Static method in class org.workflowsim.utils.Parameters
:   Gets the runtime file path

getSafetyParameter() - Method in class org.cloudbus.cloudsim.power.PowerVmAllocationPolicyMigrationInterQuartileRange
:   Gets the safety parameter.

getSafetyParameter() - Method in class org.cloudbus.cloudsim.power.PowerVmAllocationPolicyMigrationLocalRegression


getSafetyParameter() - Method in class org.cloudbus.cloudsim.power.PowerVmAllocationPolicyMigrationMedianAbsoluteDeviation
:   Gets the safety parameter.

getSavedAllocation() - Method in class org.cloudbus.cloudsim.power.PowerVmAllocationPolicyMigrationAbstract
:   Gets the saved allocation.

getScheduledList() - Method in class org.workflowsim.scheduler.BaseScheduler
:   Gets the scheduled job list

getScheduledList() - Method in interface org.workflowsim.scheduler.SchedulerInterface
:   Gets the scheduled jobs.

getScheduler(int) - Method in class org.workflowsim.WorkflowEngine
:   Gets the scheduler .

getSchedulerId(int) - Method in class org.workflowsim.WorkflowEngine
:   Gets the scheduler id list.

getSchedulerIds() - Method in class org.workflowsim.WorkflowEngine
:   Gets the scheduler id.

getSchedulerMode() - Static method in class org.workflowsim.utils.Parameters
:   Gets the scheduling method

getSchedulers() - Method in class org.workflowsim.WorkflowEngine
:   Gets the schedulers.

getSchedulingInterval() - Method in class org.cloudbus.cloudsim.Datacenter
:   Gets the scheduling interval.

getSchedulingInterval() - Method in class org.cloudbus.cloudsim.power.PowerVm
:   Gets the scheduling interval.

getSchedulingInterval() - Method in class org.cloudbus.cloudsim.power.PowerVmAllocationPolicyMigrationLocalRegression
:   Gets the scheduling interval.

getSchedulingInterval() - Method in class org.cloudbus.cloudsim.UtilizationModelPlanetLabInMemory
:   Gets the scheduling interval.

getSimulationCalendar() - Static method in class org.cloudbus.cloudsim.core.CloudSim
:   Gets a new copy of initial simulation Calendar.

getSize() - Method in class org.cloudbus.cloudsim.File
:   Gets the file size (in MBytes)

getSize() - Method in class org.cloudbus.cloudsim.InfoPacket
:   Gets the size of this packet.

getSize() - Method in interface org.cloudbus.cloudsim.Packet
:   Returns the size of this packet

getSize() - Method in class org.cloudbus.cloudsim.Vm
:   Gets the amount of storage.

getSizeInByte() - Method in class org.cloudbus.cloudsim.File
:   Gets the file size (in bytes)

getSlaMetrics(List<Vm>) - Static method in class org.cloudbus.cloudsim.examples.power.Helper
:   Gets the sla metrics.

getSlaTimePerActiveHost(List<Host>) - Static method in class org.cloudbus.cloudsim.examples.power.Helper
:   Gets the sla time per active host.

getSlaTimePerHost(List<Host>) - Static method in class org.cloudbus.cloudsim.examples.power.Helper
:   Gets the sla time per host.

getSource() - Method in class org.cloudbus.cloudsim.core.SimEvent
:   Get the unique id number of the entity which scheduled this event.

getSrcId() - Method in class org.cloudbus.cloudsim.InfoPacket
:   Gets the id of the entity that sent out this packet.

getSrcId() - Method in interface org.cloudbus.cloudsim.Packet
:   Returns the ID of the source of this packet.

getSrcNodeID() - Method in class org.cloudbus.cloudsim.network.TopologicalLink
:   returns the node-ID from the SrcNode

getStartTime() - Method in class org.cloudbus.cloudsim.ResCloudlet
:   Gets the Cloudlet or reservation start time.

getState() - Method in class org.cloudbus.cloudsim.core.SimEntity
:   Gets the state.

getState() - Method in class org.workflowsim.CondorVM
:   Gets the state of the task

getStateHistory() - Method in class org.cloudbus.cloudsim.HostDynamicWorkload
:   Gets the state history.

getStateHistory() - Method in class org.cloudbus.cloudsim.Vm
:   Gets the state history.

getStaticPower() - Method in class org.cloudbus.cloudsim.power.models.PowerModelCubic
:   Gets the static power.

getStaticPower() - Method in class org.cloudbus.cloudsim.power.models.PowerModelLinear
:   Gets the static power.

getStaticPower() - Method in class org.cloudbus.cloudsim.power.models.PowerModelSqrt
:   Gets the static power.

getStaticPower() - Method in class org.cloudbus.cloudsim.power.models.PowerModelSquare
:   Gets the static power.

getStatistics(List<Double>) - Static method in class org.cloudbus.cloudsim.util.MathUtil
:   Returns descriptive statistics for the list of numbers.

getStatistics(double[]) - Static method in class org.cloudbus.cloudsim.util.MathUtil
:   Returns descriptive statistics for the array of numbers.

getStatus() - Method in class org.cloudbus.cloudsim.Cloudlet
:   Get the status of the Cloudlet.

getStatus() - Method in class org.cloudbus.cloudsim.Pe
:   Gets the status of this Pe.

getStatusString(int) - Static method in class org.cloudbus.cloudsim.Cloudlet
:   Gets the string representation of the given Cloudlet status code.

getStorage() - Method in class org.cloudbus.cloudsim.Host
:   Gets the machine storage.

getStorageList() - Method in class org.cloudbus.cloudsim.Datacenter
:   Gets the storage list.

getStorageList(String) - Static method in class org.workflowsim.utils.ReplicaCatalog
:   Gets the list of storages a file exists

getSubmissionTime() - Method in class org.cloudbus.cloudsim.Cloudlet
:   Gets the submission or arrival time of this Cloudlet from the latest CloudResource.

getSubmissionTime(int) - Method in class org.cloudbus.cloudsim.Cloudlet
:   Gets the submission or arrival time of this Cloudlet in the given CloudResource ID.

getSubmittime() - Method in class org.cloudbus.cloudsim.network.datacenter.NetworkCloudlet


getSwitchedOffHosts() - Method in class org.cloudbus.cloudsim.power.PowerVmAllocationPolicyMigrationAbstract
:   Gets the switched off host.

getTag() - Method in class org.cloudbus.cloudsim.core.SimEvent
:   Get the user-defined tag of this event.

getTag() - Method in class org.cloudbus.cloudsim.InfoPacket
:   Gets this packet tag.

getTag() - Method in interface org.cloudbus.cloudsim.Packet
:   Gets this packet tag

getTask2Job() - Method in class org.workflowsim.clustering.BasicClustering
:   Gets the map from task to job

getTaskFiles() - Method in class org.workflowsim.clustering.BasicClustering
:   Gets the files

getTaskFiles() - Method in interface org.workflowsim.clustering.ClusteringInterface
:   get all the task files.

getTaskList() - Method in class org.workflowsim.clustering.BasicClustering
:   Gets the task list

getTaskList() - Method in interface org.workflowsim.clustering.ClusteringInterface
:   get task list.

getTaskList() - Method in class org.workflowsim.clustering.TaskSet
:   Gets task list

getTaskList() - Method in class org.workflowsim.ClusteringEngine
:   Gets the task list.

getTaskList() - Method in class org.workflowsim.Job
:   Gets the list of tasks in this job

getTaskList() - Method in class org.workflowsim.planning.BasePlanner
:   Gets the task list.

getTaskList() - Method in interface org.workflowsim.planning.PlannerInterface
:   Gets the task list.

getTaskList() - Method in class org.workflowsim.WorkflowParser
:   Gets the task list

getTaskList() - Method in class org.workflowsim.WorkflowPlanner
:   Gets the task list.

getTaskMap() - Method in class org.workflowsim.clustering.balancing.methods.BalancingMethod
:   Gets the task map

getTaskReceivedList() - Method in class org.workflowsim.ClusteringEngine
:   Gets the task received list.

getTaskSubmittedList() - Method in class org.workflowsim.ClusteringEngine
:   Gets the tasks submitted list.

getTime() - Method in class org.cloudbus.cloudsim.HostStateHistoryEntry
:   Gets the time.

getTime() - Method in class org.cloudbus.cloudsim.VmStateHistoryEntry
:   Gets the time.

getTimeHistory() - Method in class org.cloudbus.cloudsim.power.PowerVmAllocationPolicyMigrationAbstract
:   Gets the time history.

getTimesBeforeHostShutdown(List<Host>) - Static method in class org.cloudbus.cloudsim.examples.power.Helper
:   Gets the times before host shutdown.

getTimesBeforeVmMigration(List<Vm>) - Static method in class org.cloudbus.cloudsim.examples.power.Helper
:   Gets the times before vm migration.

getTimeZone() - Method in class org.cloudbus.cloudsim.DatacenterCharacteristics
:   Gets the time zone.

getTotalAllocatedMips() - Method in class org.cloudbus.cloudsim.provisioners.PeProvisioner
:   Gets the total allocated MIPS.

getTotalAllocatedMipsForVm(Vm) - Method in class org.cloudbus.cloudsim.Host
:   Gets the total allocated MIPS for a VM over all the PEs.

getTotalAllocatedMipsForVm(Vm) - Method in class org.cloudbus.cloudsim.provisioners.PeProvisioner
:   Gets total allocated MIPS for a given VM for all PEs.

getTotalAllocatedMipsForVm(Vm) - Method in class org.cloudbus.cloudsim.provisioners.PeProvisionerSimple


getTotalAllocatedMipsForVm(Vm) - Method in class org.cloudbus.cloudsim.VmScheduler
:   Gets the total allocated MIPS for a VM over all the PEs.

getTotalCurrentAllocatedMipsForCloudlet(ResCloudlet, double) - Method in class org.cloudbus.cloudsim.CloudletScheduler
:   Gets the total current allocated mips for cloudlet.

getTotalCurrentAllocatedMipsForCloudlet(ResCloudlet, double) - Method in class org.cloudbus.cloudsim.CloudletSchedulerDynamicWorkload
:   Gets the current mips.

getTotalCurrentAllocatedMipsForCloudlet(ResCloudlet, double) - Method in class org.cloudbus.cloudsim.CloudletSchedulerSpaceShared


getTotalCurrentAllocatedMipsForCloudlet(ResCloudlet, double) - Method in class org.cloudbus.cloudsim.CloudletSchedulerTimeShared


getTotalCurrentAllocatedMipsForCloudlet(ResCloudlet, double) - Method in class org.cloudbus.cloudsim.network.datacenter.NetworkCloudletSpaceSharedScheduler


getTotalCurrentAvailableMipsForCloudlet(ResCloudlet, List<Double>) - Method in class org.cloudbus.cloudsim.CloudletScheduler
:   Gets the total current mips for the Cloudlet.

getTotalCurrentAvailableMipsForCloudlet(ResCloudlet, List<Double>) - Method in class org.cloudbus.cloudsim.CloudletSchedulerDynamicWorkload
:   Gets the total current mips for the clouddlet.

getTotalCurrentAvailableMipsForCloudlet(ResCloudlet, List<Double>) - Method in class org.cloudbus.cloudsim.CloudletSchedulerSpaceShared


getTotalCurrentAvailableMipsForCloudlet(ResCloudlet, List<Double>) - Method in class org.cloudbus.cloudsim.CloudletSchedulerTimeShared


getTotalCurrentAvailableMipsForCloudlet(ResCloudlet, List<Double>) - Method in class org.cloudbus.cloudsim.network.datacenter.NetworkCloudletSpaceSharedScheduler


getTotalCurrentMips() - Method in class org.cloudbus.cloudsim.CloudletSchedulerDynamicWorkload
:   Gets the total current mips.

getTotalCurrentRequestedMipsForCloudlet(ResCloudlet, double) - Method in class org.cloudbus.cloudsim.CloudletScheduler
:   Gets the total current requested mips for cloudlet.

getTotalCurrentRequestedMipsForCloudlet(ResCloudlet, double) - Method in class org.cloudbus.cloudsim.CloudletSchedulerDynamicWorkload
:   Gets the current mips.

getTotalCurrentRequestedMipsForCloudlet(ResCloudlet, double) - Method in class org.cloudbus.cloudsim.CloudletSchedulerSpaceShared


getTotalCurrentRequestedMipsForCloudlet(ResCloudlet, double) - Method in class org.cloudbus.cloudsim.CloudletSchedulerTimeShared


getTotalCurrentRequestedMipsForCloudlet(ResCloudlet, double) - Method in class org.cloudbus.cloudsim.network.datacenter.NetworkCloudletSpaceSharedScheduler


getTotalMips() - Method in class org.cloudbus.cloudsim.CloudletSchedulerDynamicWorkload
:   Gets the total mips.

getTotalMips() - Method in class org.cloudbus.cloudsim.Host
:   Gets the total mips.

getTotalMips(List<T>) - Static method in class org.cloudbus.cloudsim.lists.PeList
:   Gets total MIPS Rating for all PEs.

getTotalResponseTime() - Method in class org.cloudbus.cloudsim.InfoPacket
:   Gets the total time that this packet has spent in the network.

getTotalUtilizationOfCpu(double) - Method in class org.cloudbus.cloudsim.CloudletScheduler
:   Get utilization created by all cloudlets.

getTotalUtilizationOfCpu(double) - Method in class org.cloudbus.cloudsim.CloudletSchedulerDynamicWorkload
:   Get utilization created by all cloudlets.

getTotalUtilizationOfCpu(double) - Method in class org.cloudbus.cloudsim.CloudletSchedulerSpaceShared
:   Get utilization created by all cloudlets.

getTotalUtilizationOfCpu(double) - Method in class org.cloudbus.cloudsim.CloudletSchedulerTimeShared
:   Get utilization created by all cloudlets.

getTotalUtilizationOfCpu(double) - Method in class org.cloudbus.cloudsim.network.datacenter.NetworkCloudletSpaceSharedScheduler
:   Get utilization created by all cloudlets.

getTotalUtilizationOfCpu(double) - Method in class org.cloudbus.cloudsim.Vm
:   Get utilization created by all clouddlets running on this VM.

getTotalUtilizationOfCpuMips(double) - Method in class org.cloudbus.cloudsim.Vm
:   Get utilization created by all cloudlets running on this VM in MIPS.

getTransactionTime() - Method in class org.cloudbus.cloudsim.File
:   Gets the last transaction time of this file (in second).

getTricubeBisquareWeigts(double[]) - Static method in class org.cloudbus.cloudsim.util.MathUtil
:   Gets the tricube bisquare weigts.

getTricubeWeigts(int) - Static method in class org.cloudbus.cloudsim.util.MathUtil
:   Gets the tricube weigts.

getType() - Method in class org.cloudbus.cloudsim.core.SimEvent
:   The internal type

getType() - Method in class org.cloudbus.cloudsim.File
:   Gets this file type

getType() - Method in class org.cloudbus.cloudsim.FileAttribute
:   Gets this file type

getType() - Method in class org.workflowsim.Task
:   Gets the type of the task

getUid() - Method in class org.cloudbus.cloudsim.ResCloudlet
:   Get unique string identificator of the VM.

getUid() - Method in class org.cloudbus.cloudsim.Vm
:   Get unique string identificator of the VM.

getUid(int, int) - Static method in class org.cloudbus.cloudsim.Vm
:   Generate unique string identificator of the VM.

getUnderAllocatedMips() - Method in class org.cloudbus.cloudsim.CloudletSchedulerDynamicWorkload
:   Gets the under allocated mips.

getUnderUtilizedHost(Set<? extends Host>) - Method in class org.cloudbus.cloudsim.power.PowerVmAllocationPolicyMigrationAbstract
:   Gets the under utilized host.

getUsedBw() - Method in class org.cloudbus.cloudsim.provisioners.BwProvisioner
:   Gets the amount of used BW in the host.

getUsedPes() - Method in class org.cloudbus.cloudsim.network.datacenter.NetworkVmAllocationPolicy
:   Gets the used pes.

getUsedPes() - Method in class org.cloudbus.cloudsim.VmAllocationPolicySimple
:   Gets the used pes.

getUsedRam() - Method in class org.cloudbus.cloudsim.provisioners.RamProvisioner
:   Gets the amount of used RAM in the host.

getUserId() - Method in class org.cloudbus.cloudsim.Cloudlet
:   Gets the user or owner ID of this Cloudlet.

getUserId() - Method in class org.cloudbus.cloudsim.ResCloudlet
:   Gets the user or owner of this Cloudlet.

getUserId() - Method in class org.cloudbus.cloudsim.Vm
:   Gets the ID of the owner of the VM.

getUtilization() - Method in class org.cloudbus.cloudsim.provisioners.PeProvisioner
:   Gets the utilization of the Pe in percents.

getUtilization(double) - Method in interface org.cloudbus.cloudsim.UtilizationModel
:   Returns utilization in percents according to the time.

getUtilization(double) - Method in class org.cloudbus.cloudsim.UtilizationModelFull


getUtilization(double) - Method in class org.cloudbus.cloudsim.UtilizationModelNull


getUtilization(double) - Method in class org.cloudbus.cloudsim.UtilizationModelPlanetLabInMemory


getUtilization(double) - Method in class org.cloudbus.cloudsim.UtilizationModelStochastic


getUtilizationHistory() - Method in class org.cloudbus.cloudsim.power.PowerHostUtilizationHistory
:   Gets the host utilization history.

getUtilizationHistory() - Method in class org.cloudbus.cloudsim.power.PowerVm
:   Gets the utilization history.

getUtilizationHistory() - Method in class org.cloudbus.cloudsim.power.PowerVmAllocationPolicyMigrationAbstract
:   Gets the utilization history.

getUtilizationMad() - Method in class org.cloudbus.cloudsim.power.PowerVm
:   Gets the utilization MAD in MIPS.

getUtilizationMatrix(List<PowerVm>) - Method in class org.cloudbus.cloudsim.power.PowerVmSelectionPolicyMaximumCorrelation
:   Gets the utilization matrix.

getUtilizationMean() - Method in class org.cloudbus.cloudsim.power.PowerVm
:   Gets the utilization mean in percents.

getUtilizationMips() - Method in class org.cloudbus.cloudsim.HostDynamicWorkload
:   Gets the utilization mips.

getUtilizationModelBw() - Method in class org.cloudbus.cloudsim.Cloudlet
:   Gets the utilization model bw.

getUtilizationModelCpu() - Method in class org.cloudbus.cloudsim.Cloudlet
:   Gets the utilization model cpu.

getUtilizationModelRam() - Method in class org.cloudbus.cloudsim.Cloudlet
:   Gets the utilization model ram.

getUtilizationOfBw(double) - Method in class org.cloudbus.cloudsim.Cloudlet
:   Gets the utilization of bw.

getUtilizationOfBw() - Method in class org.cloudbus.cloudsim.HostDynamicWorkload
:   Gets the utilization of bw.

getUtilizationOfCpu(double) - Method in class org.cloudbus.cloudsim.Cloudlet
:   Gets the total utilization of cpu.

getUtilizationOfCpu() - Method in class org.cloudbus.cloudsim.HostDynamicWorkload
:   Get current utilization of CPU in percentage.

getUtilizationOfCpuMips() - Method in class org.cloudbus.cloudsim.HostDynamicWorkload
:   Get current utilization of CPU in MIPS.

getUtilizationOfCpuMips(PowerHost) - Method in class org.cloudbus.cloudsim.power.PowerVmAllocationPolicyMigrationAbstract
:   Gets the utilization of the CPU in MIPS for the current potentially allocated VMs.

getUtilizationOfRam(double) - Method in class org.cloudbus.cloudsim.Cloudlet
:   Gets the utilization of memory.

getUtilizationOfRam() - Method in class org.cloudbus.cloudsim.HostDynamicWorkload
:   Gets the utilization of memory.

getUtilizationThreshold() - Method in class org.cloudbus.cloudsim.power.PowerVmAllocationPolicyMigrationStaticThreshold
:   Gets the utilization threshold.

getUtilizationVariance() - Method in class org.cloudbus.cloudsim.power.PowerVm
:   Gets the utilization variance in MIPS.

getVersion() - Static method in class org.workflowsim.utils.Parameters
:   Gets the version

getVm(int, int) - Method in class org.cloudbus.cloudsim.Host
:   Returns a VM object.

getVmAllocationPolicy() - Method in class org.cloudbus.cloudsim.Datacenter
:   Gets the vm allocation policy.

getVmAllocationPolicy(String, String, String) - Method in class org.cloudbus.cloudsim.examples.power.RunnerAbstract
:   Gets the vm allocation policy.

getVmId() - Method in class org.cloudbus.cloudsim.Cloudlet
:   Gets the ID of the VM that will run this Cloudlet.

getVmList() - Method in class org.cloudbus.cloudsim.Datacenter
:   Gets the vm list.

getVmList() - Method in class org.cloudbus.cloudsim.DatacenterBroker
:   Gets the vm list.

getVmList() - Method in class org.cloudbus.cloudsim.examples.CloudSimExample8.GlobalBroker


getVmList() - Method in class org.cloudbus.cloudsim.Host
:   Gets the vm list.

getVmList() - Method in class org.cloudbus.cloudsim.network.datacenter.NetDatacenterBroker
:   Gets the vm list.

getVmList() - Method in class org.workflowsim.planning.BasePlanner
:   Gets the vm list

getVmList() - Method in interface org.workflowsim.planning.PlannerInterface
:   Gets the vm list.

getVmList() - Method in class org.workflowsim.scheduler.BaseScheduler
:   Gets the vm list

getVmList() - Method in interface org.workflowsim.scheduler.SchedulerInterface
:   Gets the vm list.

getVmList() - Method in class org.workflowsim.WorkflowEngine
:   Gets the vm list.

getVmm() - Method in class org.cloudbus.cloudsim.DatacenterCharacteristics
:   Gets the VMM in use in the datacenter.

getVmm() - Method in class org.cloudbus.cloudsim.Vm
:   Gets the VMM.

getVmNum() - Static method in class org.workflowsim.utils.Parameters
:   Gets the vm number

getVmsAcks() - Method in class org.cloudbus.cloudsim.DatacenterBroker
:   Gets the vms acks.

getVmsAcks() - Method in class org.cloudbus.cloudsim.network.datacenter.NetDatacenterBroker
:   Gets the vms acks.

getVmScheduler() - Method in class org.cloudbus.cloudsim.Host
:   Gets the VM scheduler.

getVmsCreatedList() - Method in class org.cloudbus.cloudsim.DatacenterBroker
:   Gets the vm list.

getVmsCreatedList() - Method in class org.cloudbus.cloudsim.network.datacenter.NetDatacenterBroker
:   Gets the vm list.

getVmsDestroyed() - Method in class org.cloudbus.cloudsim.DatacenterBroker
:   Gets the vms destroyed.

getVmsDestroyed() - Method in class org.cloudbus.cloudsim.network.datacenter.NetDatacenterBroker
:   Gets the vms destroyed.

getVmSelectionPolicy(String) - Method in class org.cloudbus.cloudsim.examples.power.RunnerAbstract
:   Gets the vm selection policy.

getVmSelectionPolicy() - Method in class org.cloudbus.cloudsim.power.PowerVmAllocationPolicyMigrationAbstract
:   Gets the vm selection policy.

getVmsMigratingIn() - Method in class org.cloudbus.cloudsim.Host
:   Gets the vms migrating in.

getVmsMigratingIn() - Method in class org.cloudbus.cloudsim.VmScheduler
:   Gets the vms migrating in.

getVmsMigratingOut() - Method in class org.cloudbus.cloudsim.VmScheduler
:   Gets the vms in migration.

getVmsRequested() - Method in class org.cloudbus.cloudsim.DatacenterBroker
:   Gets the vms requested.

getVmsRequested() - Method in class org.cloudbus.cloudsim.network.datacenter.NetDatacenterBroker
:   Gets the vms requested.

getVmsToDatacentersMap() - Method in class org.cloudbus.cloudsim.DatacenterBroker
:   Gets the vms to datacenters map.

getVmsToDatacentersMap() - Method in class org.cloudbus.cloudsim.network.datacenter.NetDatacenterBroker
:   Gets the vms to datacenters map.

getVmsToMigrateFromHosts(List<PowerHostUtilizationHistory>) - Method in class org.cloudbus.cloudsim.power.PowerVmAllocationPolicyMigrationAbstract
:   Gets the vms to migrate from hosts.

getVmsToMigrateFromUnderUtilizedHost(PowerHost) - Method in class org.cloudbus.cloudsim.power.PowerVmAllocationPolicyMigrationAbstract
:   Gets the vms to migrate from under utilized host.

getVmTable() - Method in class org.cloudbus.cloudsim.network.datacenter.NetworkVmAllocationPolicy
:   Gets the vm table.

getVmTable() - Method in class org.cloudbus.cloudsim.power.PowerVmAllocationPolicyAbstract
:   Gets the vm table.

getVmTable() - Method in class org.cloudbus.cloudsim.VmAllocationPolicySimple
:   Gets the vm table.

getVmToMigrate(PowerHost) - Method in class org.cloudbus.cloudsim.power.PowerVmSelectionPolicy
:   Gets the vms to migrate.

getVmToMigrate(PowerHost) - Method in class org.cloudbus.cloudsim.power.PowerVmSelectionPolicyMaximumCorrelation


getVmToMigrate(PowerHost) - Method in class org.cloudbus.cloudsim.power.PowerVmSelectionPolicyMinimumMigrationTime


getVmToMigrate(PowerHost) - Method in class org.cloudbus.cloudsim.power.PowerVmSelectionPolicyMinimumUtilization


getVmToMigrate(PowerHost) - Method in class org.cloudbus.cloudsim.power.PowerVmSelectionPolicyRandomSelection


getWaitingTime() - Method in class org.cloudbus.cloudsim.Cloudlet
:   Gets the waiting time of this cloudlet executed on a resource.

getWallClockTime() - Method in class org.cloudbus.cloudsim.Cloudlet
:   Gets the time of this Cloudlet resides in the latest CloudResource (from arrival time until
    departure time).

getWallClockTime(int) - Method in class org.cloudbus.cloudsim.Cloudlet
:   Gets the time of this Cloudlet resides in a given CloudResource ID (from arrival time until
    departure time).

getWEDDelay() - Method in class org.workflowsim.utils.OverheadParameters
:   Gets the workflow engine delay

getWEDDelay(List) - Method in class org.workflowsim.utils.OverheadParameters
:   Gets the workflow engine delay for a particular job based on the
    depth(level)

getWEDInterval() - Method in class org.workflowsim.utils.OverheadParameters
:   Gets the interval

getWorkflowEngine() - Method in class org.workflowsim.ClusteringEngine
:   returns the WorkflowEngine

getWorkflowEngine() - Method in class org.workflowsim.WorkflowPlanner
:   Gets the workflow engine

getWorkflowEngineId() - Method in class org.workflowsim.ClusteringEngine
:   returns the WorkflowEngineId

getWorkflowEngineId() - Method in class org.workflowsim.WorkflowPlanner
:   Gets the workflow engine id

getWorkflowParser() - Method in class org.workflowsim.WorkflowPlanner
:   Gets the workflow parser

graph - Static variable in class org.cloudbus.cloudsim.NetworkTopology


GraphReaderBrite - Class in org.cloudbus.cloudsim.network
:   This class is just an file-reader for the special brite-format! the brite-file is structured as
    followed: Node-section: NodeID, xpos, ypos, indegree, outdegree, ASid, type(router/AS)
    Edge-section: EdgeID, fromNode, toNode, euclideanLength, linkDelay, linkBandwith, AS\_from, AS\_to,
    type

GraphReaderBrite() - Constructor for class org.cloudbus.cloudsim.network.GraphReaderBrite


GraphReaderIF - Interface in org.cloudbus.cloudsim.network
:   This interface abstracts an reader for different graph-file-formats

A B C D E F G H I J L M N O P Q R S T U V W Z

- Overview
- Package
- Class
- Use
- Tree
- Deprecated
- Index
- Help

- Prev Letter
- Next Letter

- Frames
- No Frames

- All Classes
